# Targeting FZD6 creates therapeutically actionable vulnerabilities for advanced prostate cancer

**DOI:** 10.1038/s41388-025-03631-6

**Published:** 2025-11-24

**Authors:** Yongtao Li, Zhicheng Zhou, Yiqun Zhang, Deyong Jia, Ding Wang, Mary C. Reiger, Chad J. Creighton, Peter S. Nelson, Eva Corey, Colm Morrissey, Li Xin

**Affiliations:** 1 Department of Urology, University of Washington, Seattle, WA USA; 2 Dan L. Duncan Comprehensive Cancer Center, Baylor College of Medicine, Houston, TX USA; 3 Institute of Stem Cell and Regenerative Medicine, University of Washington, Seattle, WA USA; 4 Department of Medicine, Baylor College of Medicine, Houston, TX USA; 5 Division of Human Biology, Fred Hutchinson Cancer Center, Seattle, WA USA

**Keywords:** Prostate cancer, Cancer therapeutic resistance

## Abstract

Wnt signaling is a complex pathway consisting of numerous ligands and frizzled (FZD) receptors. These signaling components are widely expressed in human prostate tissues and often undergo upregulation or mutation in advanced prostate cancers. Enhanced Wnt signaling promotes prostate cancer cell proliferation, metastasis, and resistance to therapy. However, targeting pan-Wnt signaling poses challenges due to tissue toxicity. We show that *FZD6* is the most highly expressed and frequently amplified Wnt receptor in advanced human prostate cancers. Knockdown of *FZD6* suppresses both in vitro and in vivo growth of various prostate cancer cell lines and patient-derived xenograft models. *FZD6* knockdown impairs DNA double-strand break (DSB) repair, as determined by both resolution of γH2AX foci and DNA DSB repair reporter assays. Mechanistically, FZD6 knockdown-induced growth suppression is linked to reduced activities of SRC kinase and STAT3, while DNA damage repair deficiency is mediated through WEE1 downregulation via PLK1. Knockdown of *FZD6* enhances the therapeutic efficacy of genotoxic agents for prostate cancer cells. A kinome-wide CRISPR-Cas9 knockout screen reveals that *FZD6* inhibition sensitizes prostate cancer cells to the inhibition of PKMYT1, a WEE kinase family member. Collectively, we demonstrate that targeting a single FZD receptor highly expressed in prostate cancers can yield significant therapeutic efficacy, and uncover therapeutic vulnerabilities associated with *FZD6* inhibition.

## Introduction

Prostate cancer is the second leading cause of cancer-related death in men in the United States. Prostate cancer initially responds to anti-androgen therapies (castration sensitive prostate cancer, CSPC) but usually recurs as a disease state termed castration resistant prostate cancer (CRPC). Despite the application of the more potent new-generation androgen receptor signaling inhibitors (ARSI), CRPC continues to develop resistance through various mechanisms that include lineage plasticity [1–3] and evolves into different lethal subtypes that include neuroendocrine prostate cancer (NEPC, expressing neuroendocrine cell markers but not androgen receptor) and double negative prostate cancer (DNPC, expressing neither androgen receptor nor neuroendocrine marker) [4]. Effective treatment for these lethal subtypes of prostate cancer is still lacking, highlighting an urgent need to identify new therapeutic targets.

Wnt signaling is a fundamental but complex growth control pathway that drives developmental processes, repair mechanisms and key aspects of neoplastic growth [5, 6]. There are 19 Wnt ligands in mammalian cells. These ligands differentially bind to 10 distinct frizzled (FZD) receptors on the membrane together with other coreceptors to activate the canonical signaling (via β-catenin) and noncanonical signaling (the planar cell polarity and Ca^2+^ pathways). Mutations in the Wnt signaling components such as β-catenin and APC etc. are rare in primary prostate cancers, but occur in CRPCs [7, 8]. In addition, noncanonical Wnt ligand Wnt5a is also highly expressed in circulating prostate tumor cells [9]. Finally, microenvironment-mediated signaling can increase the frequency of Wnt pathway activation as a driver of CRPC [10]. These observations support the critical role of both canonical and noncanonical Wnt signaling in prostate cancer progression.

Functional studies confirmed that elevated Wnt signaling in prostate tumor cells induces proliferation, metastasis, and neuroendocrine differentiation [11–17]. However, insufficient work has been performed to investigate the potential of targeting Wnt signaling to treat advanced prostate cancer. Previous studies applied natural Wnt inhibitors such as DKKs, WIF-1, and sFRPs or small-molecule pan-Wnt inhibitors to target Wnt signaling non-selectively [18]. Unfortunately, these approaches cause dose-limiting toxicities due to depletion of intestinal stem cells or bone loss. Therefore, identification of agents that block only portions of Wnt signaling that are distinctly presented in specific organs and cell types is of great interest. We reason that targeting just the FZD receptor(s) highly expressed in prostate cancer will be sufficient to achieve therapeutic responses, while alleviating tissue toxicity. In this study, we show that *FZD6* is the most highly expressed and frequently amplified Wnt receptor in advanced prostate cancer and provide evidence that targeting FZD6 in various aggressive subtypes of prostate cancers suppresses tumor growth, impairs DNA damage repair response, and enhances the therapeutic efficacy of cisplatin in a preclinical prostate cancer model.

## Materials and methods

### Mice

All animals used in this study received humane care in compliance with the principles stated in the Guide for the Care and Use of Laboratory Animals, NIH Publication, 1996 edition, and the protocol was approved by the Institutional Animal Care Committees of University of Washington (protocol no. 4431-01). The NOD/SCID mice were purchased from Charles River (Wilmington, MA). Experimental mice were randomly grouped, and experiments were set up and analyzed in a blind manner whenever possible.

### Doxycycline, cisplatin, and BrdU treatment

Doxycycline (Sigma-Aldrich, St. Louis, MO) was dissolved in drinking water to a final concentration of 2 mg/ml supplemented with 0.5% sucrose. Cisplatin (MedChemExpress, Monmouth Junction, NJ) was dissolved in saline and administered i.p. at a dosage of 2.5 mg/kg every three days till the experiments were terminated. BrdU (Sigma-Aldrich, St. Louis, MO) (80 mg/kg) was administrated to experimental mice once at 4–8 h before they were euthanized.

### PDX models

All patient-derived xenograft (PDX) experiments were approved by the University of Washington Institutional Animal Care and Use Committee (protocol no. 4431-01). LuCaP PDX lines were established from specimens acquired at either radical prostatectomy or at autopsy, implanted, and maintained by serial passage in immune compromised male mice as previously described [19].

### Cell culture

All cells were maintained at 37 °C in humidified Steri-Cult CO2 incubators (Thermo Fisher Scientific, Waltham, MA). RWPE, PC3 and DU145 (gifts from M Ittamnn at Baylor College of Medicine), WPMY-1 (gift from Chawnshang Chang at University of Rochester), LNCaP, C4-2, C4-2B, 22Rv1, and LN95 cells (gifts from S. Plymate at University of Washington) were maintained in RPMI-1640 (Thermo Fisher Scientific, Waltham, MA) supplemented with 10% fetal bovine serum (GenDEPOT, Katy, TX). All cell lines were validated by short tandem repeat analysis using ATCC reference genomes and were routinely tested for mycoplasma using the MycoFluor Mycoplasma Detection Kit (Invitrogen, Carlsbad, CA). The chemicals used to treat cells including lunresertib (0 - 8 µM), Cycloheximide (50 µg/ml), MG-132 (10 µM), and etoposide (2 µM) were all from MedChemExpress (Monmouth Junction, NJ).

### MTT and Click-iT EdU cell proliferation assays

Cells were plated into flat-bottomed 96-well plates, 1000–3000 cells per well, and cultured in full growth medium for 3-7 days. For drug response analysis, cells were cultured overnight before drugs of specified concentrations were added and cells were incubated for 3-5 days. At the end of experiments, 20 µL of MTT (5 mg/ml) (Sigma-Aldrich, St. Louis, MO) reagent was added to each well, and the plates were incubated at 37 °C for additional 4 h. Following incubation, the insoluble formazan crystals were dissolved into 200 µl DMSO. The spectrophotometric absorbance of the samples was detected by using an iMark microplate reader (Bio-Rad, Hercules, CA) at 560 nm with a reference wavelength of 630 nm. For Click-iT EdU Cell Proliferation Assay (Thermo Fisher Scientific, Waltham, MA), cells were treated with EdU for 2-4 h before analysis.

### RNA purification and quantitative PCR (qPCR) analysis

Total RNA was extracted using a NucleoSpin RNA Plus kit (Macherey-Nagel, Bethlehem, PA) and reverse transcribed using an iScript cDNA Synthesis Kit (Bio-Rad, Hercules, CA). Primers used in the qPCR experiments are listed in the Supplementary Table 2. qPCR was performed using the SYBR Green Gene Expression Assays (Bio-Rad, Hercules, CA) on a QuantStudio5 Real-Time PCR system (Applied Biosystems, Foster City, CA). The expression levels of genes were normalized to that of *GAPDH*.

### Kinome-wide CRISPR-Cas9 screen

DU145 cells expressing doxycycline-inducible *FZD6* shRNA were used for the screen. The human Kinome CRISPR pooled library purchased from Addgene (catalogue# 75314) was amplified based on the protocol provided by Addgene using Endura electrocompetent cells. The lentivirus library was produced in 293T cells, filtered through a 0.45 μm cellulose membrane and concentrated with a high-speed centrifuge (20,000 rpm, 2 h). The viral titer was determined using DU145 cells by measuring puromycin resistance after infection.

Infections were set up at a 1500-fold coverage of the library. DU145 cells were infected at an m.o.i. of 0.3 with the kinome library viruses followed by puromycin selection for 4 days. The cells were divided into two portions and cultured with and without doxycycline for 7 days. The sgRNAs incorporated into the cells were amplified from genomic DNA and sequenced on a NextSeq2000 (Illumina) with read1 = 100 bp and index1 = 8 bp, yielding at least 1.8 million reads per sample index. We used the MAGeCK v0.5.9.5 software package [20] to subcommand, count, and extract sgRNA read count information from FASTQ files. MAGeCK’s test subcommand was implemented to determine sgRNA and gene rankings respectively and thereby identify candidate genes whose guides were most enriched or depleted in doxycycline treated samples.

### RNA sequencing

TruSeq Stranded mRNA Sample Preparation Kit (Illumina, San Diego, CA) was used to prepare cDNA libraries that were sequenced using HiSeq 2500 sequencer. Sequenced reads in FASTQ files were mapped to mm10 whole genome using STAR, and Fragments Per Kilobase of transcript per Million mapped reads (FPKM) were calculated using Cufflinks. Genes found differentially expressed (*p* < 0.01 by *t*-test, and minimum average fold change of 1.2, using log2-transformed values) were evaluated for enrichment of Gene Ontology (GO) gene classes, using SigTerms software. Data has been deposited at GEO: GSE276026.

### Plasmids and lentivirus production

Human FZD6 ORF was purchased from Origene (Rockville, MD). The shRNA-resistant mutant was generated using QuikChange II Site-Directed Mutagenesis Kit (Agilent, Santa Clara, CA). Wild-type and mutant *FZD6* were subcloned into FUGW lentiviral vector at XbaI site for constitutive expression or subcloned into pCW57.1 lentiviral vector (Addgene, #41393) at *NheI* and *SalI* sites for doxycycline-inducible overexpression. Scrambled shRNA, shRNAs for *FZD6* and *PLK1* were cloned into pLKO.1 (Addgene, #10878) for constitutive expression or Tet-pLKO-GFP for doxycycline-inducible expression. Tet-pLKO-GFP was made by replacing the puromycin resistant gene in the Tet-pLKO-Puro (Addgene, #21915) with a GFP-WRE cassette from the FUGW plasmid (Addgene, #14883) using *KpnI*. Lentivirus preparation, titration, and infection of human cells were performed as described previously [21]. The primers used for cloning and the targeting sequences of the *FZD6* and *PLK1* shRNAs are listed in Supplementary Table 3.

### DNA repair reporter assays

The pDRGFP (Addgene, #26475) and pimEJ5GFP (Addgene, #44076) plasmids were transfected into C4-2 cells separately using lipofectamine 3000. Stable integration of the plasmids was selected using 1.8 µg/ml puromycin. Assays were performed as previously described [22]. In brief, cells were seeded for Lipofectamine 3000 transfections with plasmids expressing I-SceI (pCBASceI, Addgene, #26477) and RFP (FU-CRW) [23]. Two days after transfection, GFP-positive cells were quantified by flow cytometry on an LSRFortessa (BD Biosciences, Franklin Lakes, NJ) and the ratio was normalized by transfection efficiency based on the percentage of RFP-expressing cells. Data were analyzed using FlowJo software (BD Biosciences, Franklin Lakes, NJ).

### Genetic engineering of human LuCaP PDX models

LuCaP PDX models were mechanically dissected into small chunks of 1–10 mm^3^ using sterile blades and incubated in 5 mg/ml collagenase type II/ advanced DMEM/F12 (1 ml per 50 mg tissue) with 10 μM of Y-27632 (STEMCELL Technologies, Vancouver, BC, Canada) at 37 °C for 3 h. Tissues were pelleted, resuspended, and incubated in chilled 0.25% Trypsin-EDTA for 5 min. Then, the tissues were pelleted, resuspended in Dispase (Invitrogen, Carlsbad, CA, 5 mg/ml) and DNase I (Roche Applied Science, Indianapolis, IN, 1 mg/mL), and pipetted vigorously to dissociate cell clumps. Dissociated cells were then passed through 70 μm cell strainers (BD Biosciences, San Jose, CA) to obtain single cells. Dissociated single cells were cultured in RPMI-1640 supplemented with 10% FBS together with lentivirus expressing GFP and the scrambled shRNA or shRNA against *FZD6* at an m.o.i of 5–10 over night. Cells were then washed with PBS and reinoculated subcutaneously into NOD/SCID male mice. Once the tumor diameter reached 1 cm, tumors were dissociated into single cells again. The cells expressing shRNAs were FACS-isolated based on their GFP positivity using Aria III (BD Biosciences, San Jose, CA) and reinoculated subcutaneously into NOD/SCID host. Once tumors regrew, the engineered PDX models were established and passaged regularly.

### Cell fractionation

DU145 cells were washed twice with PBS and scraped off from petridish in cold PBS and pelleted at 600 × *g* for 5 min. Fractionation of DU145 cell lysates was carried out using a Cytoplasmic and Nuclear Protein Extraction Kit (Boster Biological Technology, Pleasanton, CA) according to the manufacturer’s protocol. All procedures were conducted on ice or at 4 °C. The nuclear and cytoplasmic fractions were analyzed by SDS-PAGE and immunoblotting.

### Western blots

Cells or tissues were lysed in RIPA buffer (20 mM Tris-HCl, pH 7.5, 150 mM NaCl, 1 mM Na_2_EDTA, 1 mM EDTA, 1% NP-40, 1% sodium deoxycholate, 2.5 mM sodium pyrophosphate, 1 mM β-glycerophosphate, 1 mM Na_3_VO_4_) supplemented with protease cocktail inhibitors and phosphatase inhibitors (GenDEPOT, Katy, TX). The protein concentration was measured using a Bradford Assay kit (BioRad, Hercules, CA). Western blot analysis was performed with precast gradient gels (4–12%) (GenScript, Piscataway, NJ) using standard methods. Proteins were transferred onto 0.2 µm nitrocellulose membrane (Bio-Rad, Hercules, CA). Membranes were blocked in 5% non-fat milk (Santa Cruz Biotech, Dallas, TX) in PBST for 1 h at room temperature, and then incubated with specific primary antibodies overnight at 4 °C, washed in PBST, incubated with an HRP-conjugated secondary antibody (R&D Systems Inc, Minneapolis, MN) for 1 h at room temperature, and developed by the ECL reagent (Thermal Scientific, Rockford, IL). The bands were visualized by an Amersham Imager 600 (Cytiva, Wilmington DE). The details of the antibodies are listed in Supplementary Table 4.

### Statistical analysis

Data are presented as means ± s.d. or s.e.m. Student’s *t* test and one-way or two-way ANOVA with multiple comparisons were used to determine significance in two-group and multiple-group experiments, respectively. Log-rank (Mantel–Cox) test was used to compare survival rates. For all statistical tests, the two-tail *p* ≤ 0.05 level of confidence was accepted for statistical significance.

## Results

### *FZD6* is highly expressed and frequently amplified in advanced prostate cancer

**Fig. 1:**
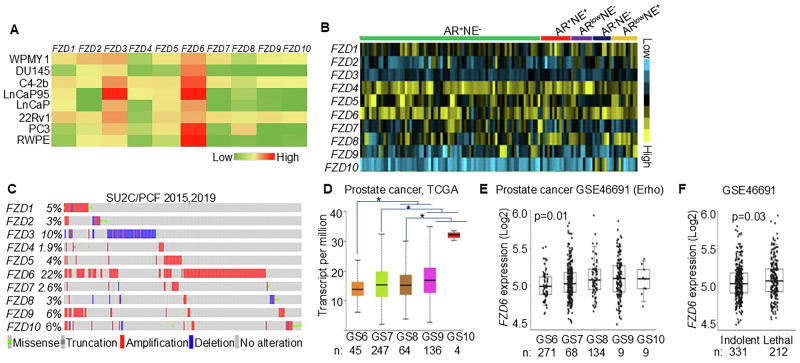
FZD6 is highly expressed and amplified in human prostate cancer and its expression inversely correlates with prostate cancer progression. **A** Heatmap of expression of Frizzled receptors in 6 prostate cell lines by qRT-PCR. **B** Heatmap of expression of Frizzled receptors in LuCaP PDX models by RNA-seq. **C** Genomic alterations in Frizzled receptors in SU2C/PCF human prostate cancer dataset. **D**, **E** Box plots show that *FZD6* expression correlations with Gleason scores in two human prostate cancer datasets. *P*-value by Pearson’s correlation. **F** Box plot shows a correlation of *FZD6* expression with lethal prostate cancers in the Erho dataset. Boxplot *P*-values by homoscedastic *t*-test. Box plots represent 5% (lower whisker), 25% (lower box), 50% (median), 75% (upper box), and 95% (upper whisker).

Wnt signaling components are widely expressed in prostate cancer [24]. To determine whether specific FZD receptors play a dominant role in prostate cancer progression, we examined the expression of all *FZD* receptors in a collection of prostate cancer cells lines. *FZD6* and *FZD3* are the most highly expressed Wnt receptors in the cancer cell lines (Fig. 1A). *FZD6* is also consistently expressed in the LuCaP PDX models representing a wide spectrum of prostate cancer genotypes and phenotypes including CRPC, DNPC, and NEPC (Fig. 1B). Alterations in all *FZD* receptors were also assessed using a cohort of metastatic prostate cancer from the Stand Up to Cancer/Prostate Cancer Foundation (SU2C/PCF) prostate cancer collection [8, 25]. *FZD6* is the most frequently amplified among all the FZD receptors (Fig. 1C). Finally, bioinformatic analysis shows that the expression level of *FZD6* positively correlates with the Gleason grade of the primary prostate cancer specimens in two independent human prostate cancer datasets (Fig. 1D, E). Higher *FZD6* expression also correlates with an adverse clinical feature in the Erho dataset (Fig. 1E). Collectively, these data show that *FZD6* expression is increased during prostate cancer progression and associate with worse outcome.

### *FZD6* downregulation suppresses growth of advanced prostate cancer in vitro and in vivo

**Fig. 2:**
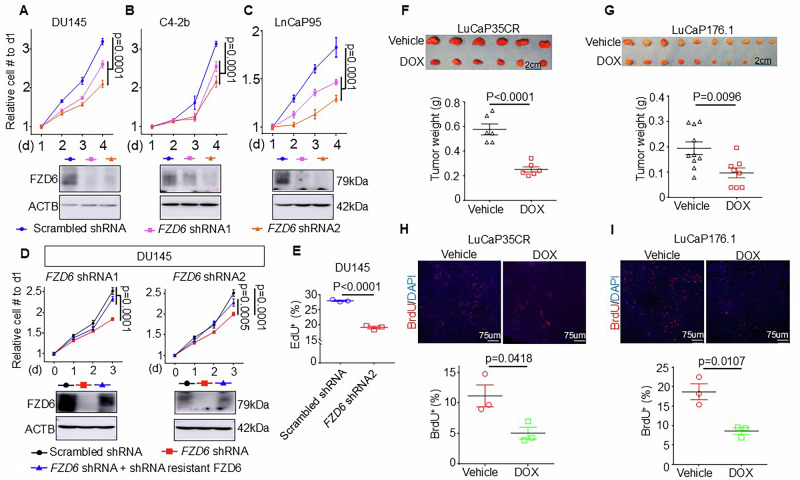
FZD6 knockdown suppresses prostate cancer growth. Growth curves of DU145 (**A**), C4-2B (**B**), and LN95 (**C**) expressing scrambled shRNA and shRNAs against *FZD6*. Dot plots show mean ± s.d. of cell numbers normalized by initial seeding numbers from triplicates. *P* values by two-way ANOVA with Dunnett’s multiple comparison test. Images below curves show expression of FZD6 by western blot. **D** Growth curves of DU145 cells expressing scrambled shRNA, shRNA against *FZD6*, and shRNA against *FZD6* together with shRNA-resistant *FZD6*. Dot plots show mean ± s.d. of cell numbers normalized by initial seeding numbers from triplicates. *P* values by two-way ANOVA with Dunnett’s multiple comparison test. Images below curves show expression of FZD6 by Western blot. **E** Dot plot shows mean ± s.d. of EdU^+^ cells in DU145 cells expressing scrambled and *FZD6* shRNAs from 3 experiments. Unpaired Student’s *t* test. **F**, **G** Doxycycline-induced *FZD6* knockdown suppresses growth of LuCaP35CR (*N* = 6) and LuCaP176 (*N* = 10 for vehicle and 8 for DOX group). Images show tumors collected from SCID/Biege hosts treated with vehicle and doxycycline (DOX). Dot plots show means ± s.d. of tumor weight. Student’s *t* test. **H**, **I** Immunostaining of BrdU. Dot plots show means ± s.e.m. of BrdU^+^ tumor cells. Each dot represents an average value of 15 random images from one xenograft. *N* = 3. Student’s *t* test.

To investigate the role of FZD6 in prostate cancer progression, we used 2 shRNAs to knock down *FZD6* in 3 representative human prostate cancer cell lines that highly express *FZD6*: the androgen receptor (AR) negative DU145, androgen-independent but AR-expressing C4-2B and LN95. Knocking down *FZD6* reduced the growth of all three lines in vitro by 18.4–34.3% (Fig. 2A–C). The specificity of the two shRNAs were confirmed by showing that re-expressing a shRNA-resistant *FZD6* transcript rescued the growth inhibitory effect of the shRNAs in the DU145 cells (Fig. 2D). *FZD6* knockdown reduced the proliferation of DU145 cells by 31.6% based on the EdU incorporation assay (Fig. 2E) but did not affect cell apoptosis as determined by the expression of cleaved PARP (Supplementary Fig. 1A). We successfully engineered two PDX models that represent CRPC (LuCaP35CR) and DNPC (LuCaP176) by infecting cells with a lentivirus that mediates the doxycycline-regulatable expression of the *FZD6* shRNA2 (Supplementary Fig. 1B). Knocking down *FZD6* inhibited the growth of the two PDX models engrafted subcutaneously in immunodeficient male NOD/SCID mice by 50.4% and 56.3%, respectively (Fig. 2F, G). Immunostaining of BrdU shows that the proliferation indices of tumor cells decreased in both models by 54% upon *FZD6* knockdown (Fig. 2H, I). Collectively, these results demonstrate that knocking down *FZD6* suppresses the growth of aggressive subtypes of prostate cancer.

### FZD6 knockdown impairs DNA double strand break repair

**Fig. 3:**
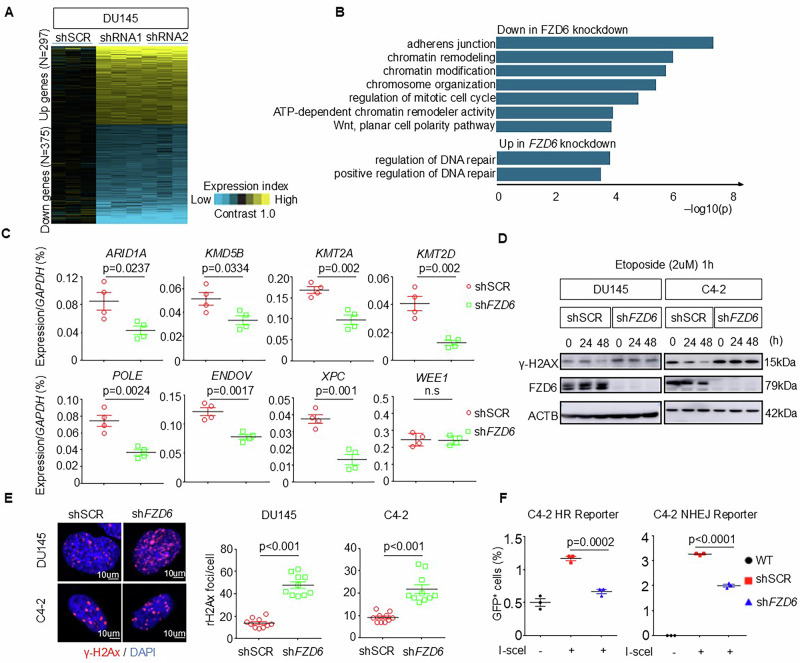
FZD6 knockdown impairs DNA damage repair. **A** Heatmap of RNA-Seq analysis of DU145 cells expressing scrambled (shSCR) shRNA and two shRNAs against *FZD6*. **B** Gene ontology analysis of RNA-Seq. **C** qRT-PCR analysis of 7 genes associated with DNA damage repair and chromatin remodeling in DU145 cells expressing scrambled (shSCR) and *FZD6* shRNA (*shFZD6*). Data represent means ± s.d. from four independent experiments. *P* values by Student’s *t* test. **D** Western blot analysis of γ-H2AX and FZD6 in DU145 and C4-2 that express scrambled (shSCR) and *FZD6* shRNAs at 0, 24, and 48 h post etoposide treatment. **E** Representative immunofluorescence images of γ-H2AX. Dot plots show means ± s.d. of γ-H2AX foci per cell. *N* = 10. **F** Dot plots show means ± s.d. of GFP^+^ C4-2 cells by flow cytometric analyses in the HR- and NHEJ- DNA double strand break repair reporter assays. Each dot shows result from one independent experiment. *P* values by One-way ANOVA with Sidak’s multiple comparison test.

To investigate how *FZD6* knockdown affects prostate cancer cell growth, we compared the gene expression profiles of DU145 cells that expressed *FZD6* shRNAs and scrambled shRNA. RNA-seq analysis revealed that 297 genes were upregulated and 375 genes were downregulated by at least 1.2-fold [(*p* < 0.01, *t*-test on log2-transcformed data)] in the DU145 cells with *FZD6* knockdown than in the control cells expressing the scrambled shRNA (Fig. 3A). In agreement with the impact of *FZD6* knockdown on cell proliferation, gene ontology analysis (Fig. 3B) showed that the genes associated with “regulation of mitotic cell cycle” were downregulated in the *FZD6* knockdown group. Genes associated with “adherens junction” and “planar cell polarity” were also downregulated, which is consistent with the role of FZD6 in noncanonical Wnt signaling. Finally, gene ontologies including “chromatin remodeling” and “chromatin modification” were decreased and the gene ontologies “regulation of DNA repair” and “regulation of double strand break repair” were increased in the *FZD6* knockdown group, implying that the DNA damage repair process was altered by the *FZD6* knockdown. QRT-PCR analysis (Fig. 3C) confirmed the downregulation of representative genes associated with chromatin organization (*ARID1A, KMD5B*, and *KMT2A/D*) and DNA repair (*POLE, ENDOV*, and *XPC*).

Next, we sought to interrogate whether *FZD6* knockdown influences DNA-damage repair. Etoposide is a chemotherapeutic drug that induces DNA double strand breaks (DSBs) by inhibiting the topoisomerase II ligase activity. Transient exposure to etoposide caused accumulation of γ-H2AX foci in DU145 and C4-2 cells, which were resolved gradually (Fig. 3D). However, the resolution of the γ-H2AX foci was delayed in the *FZD6* knockdown cells. Immunostaining shows that the average numbers of γ-H2AX foci per cell in the *FZD6* knockdown DU145 and C4-2 cells were 2.4- and 3.4-fold more than those of the control DU145 and C4-2 cells expressing scrambled shRNA, respectively, demonstrating a delayed clearance of γ-H2AX foci (Fig. 3E). These results show that *FZD6* knockdown impairs DNA damage repair. We further utilized the DR-GFP (direct repeats green fluorescent protein) and EJ5-GFP (a cassette containing a promoter that is separated from *GFP* by a *Puro* gene flanked by two I-SceI sites) fluorescence-based assays [26] to determine whether *FZD6* knockdown impairs DNA double strand break repair via homologous recombination (HR) or non-homologous end-joining (NHEJ) activity, respectively. C4-2 cells with stably integrated DR-GFP and EJ5-GFP reporters were established respectively. These cells were transfected to transiently express I-SceI, and the percentage of GFP^+^ cells was quantified. The HR and NHEJ-mediated DSB repairs were attenuated by 38.8% and 42.9%, respectively, by *FZD6* knockdown (Fig. 3F). Collectively, these results show that *FZD6* knockdown impairs DNA damage repair, which is consistent with a previous study showing that a pan-Wnt inhibitor that blocks the secretion of Wnt ligands attenuates DNA damage repair in colorectal cancer cells [27].

### *FZD6* knockdown suppresses proliferation and attenuates DNA damage repair by downregulating SRC and WEE1, respectively

**Fig. 4:**
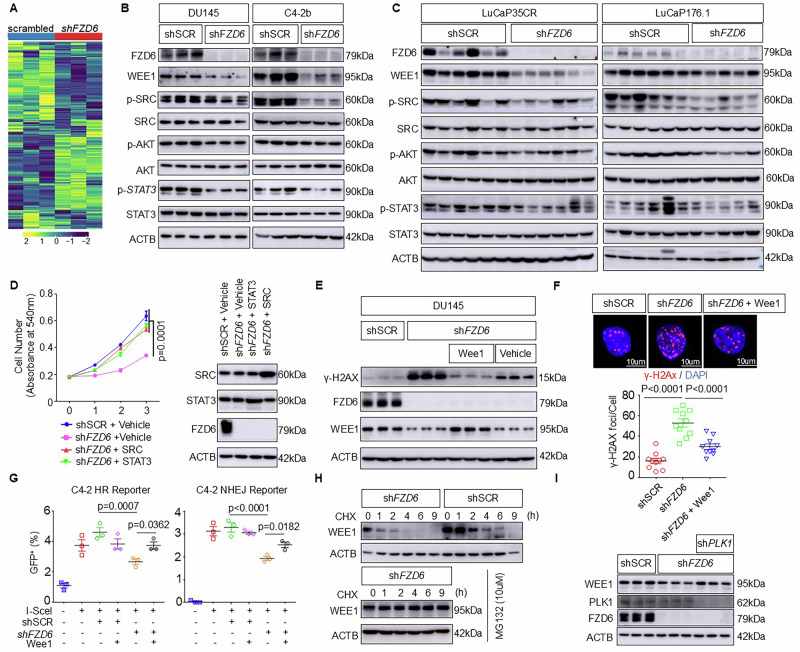
FZD6 knockdown attenuates DNA damage repair via WEE1. **A** Heatmap of reverse-phase protein array analysis of DU145 cells expressing scrambled (shSCR) shRNA and two shRNAs against *FZD6*. **B** Western blot assays of indicated proteins in DU145 and C4-2b cells expressing scrambled (shSCR) shRNA and *FZD6* shRNA (*shFZD6*). **C** Western blot assays of indicated proteins in LuCaP35CR and LuCaP176.1 expressing scrambled (shSCR) shRNA and *FZD6* shRNA (*shFZD6*). **D** MTT assay of DU145 expressing scrambled shRNA, *FZD6* shRNA, *FZD6* shRNA with SRC or STAT3. Data represent means ± s.d. of triplicates. *P* values by two-way ANOVA with Dunnett’s multiple comparison test. Western blot shows knockdown of FZD6 and ectopic expression of SRC and STAT3. **E** Western blot assay of indicated proteins in DU145 cells expressing scrambled shRNA (shSCR), and *shFZD6* with or without WEE1. Three lanes in each group represent three independent experiments. **F** Immunostaining of γH2AX in DU145 cells expressing scrambled shRNA (shSCR), sh*FZD6*, and sh*FZD6* with WEE1. Dot plot shows mean ± s.d. of γ-H2AX foci per cell. *N* = 10. *P* values by one-way ANOVA with Sidak’s multiple comparison test. **G** Dot plots show means ± s.d. of GFP^+^ C4-2 cells by flow cytometric analyses in the HR- and NHEJ- DNA double strand break repair reporter assays. Each dot shows result from one experiment. *N* = 3. *P* values by One-way ANOVA with Sidak’s multiple comparison test. **H** Western blot of WEE1 and β-actin in DU145 cells expressing scrambled (shSCR) shRNA and *FZD6* shRNA (*shFZD6*) in the presence of varying concentration of cycloheximide (CHX) with and without MG132. **I** Western blot assay of WEE1, FZD6, and β-actin in DU145 cells expressing scrambled (shSCR) shRNA, *FZD6* shRNA (*shFZD6*) and *shFZD6* together with shRNA against *PLK1* (sh*PLK1*). Three lanes for each condition represent three independent experiments.

To investigate the mechanisms by which *FZD6* knockdown impairs the growth and DNA damage repair of prostate cancer cells, we performed a reverse-phase protein array (RPPA) with 466 antibodies to identify differentially expressed proteins between the DU145 cells expressing the *FZD6* shRNAs and control scrambled shRNA. The RPPA assay identified 21 and 26 proteins that are upregulated and downregulated respectively by more than 1.25-fold in the *FZD6* knockdown DU145 cells versus control cells, respectively (Fig. 4A and Supplementary Table 1). Consistent with the tumor inhibitory effect of *FZD6* knockdown, the activities of proteins that promote tumor growth including SRC, AKT1, and STAT3 were decreased. In addition, several proteins involved in DNA damage response and genome stability (Wee1, ATM, ATM pSer1981, XRCC1, ARID1A) were also decreased upon *FZD6* knockdown. Western blot analyses confirm the downregulation of WEE1 and decreased activities of SRC, STAT3, and AKT1 by *FZD6* knockdown in DU145, C4-2B, LuCaP35CR, and LuCaP176.1 (Fig. 4B, C). Phosphorylation of S6K and 4E-BP1were both downregulated in the *FZD6* knockdown DU145 cells, further corroborating the reduced AKT activation (Supplementary Fig. 2A).

Wnt has been reported to activate SRC and STAT3 to promote cell growth and survival [28, 29]. Ectopic expressions of constitutively activated SRC or STAT3 rescued the growth suppression caused by *FZD6* knockdown (Fig. 4D), consistent with their roles in promoting cell proliferation. In contrast, overexpressing SRC or STAT3 did not rescue the delayed resolution of the H2AX foci by etoposide in the DU145 cells with *FZD6* knockdown, indicating that SRC and STAT3 do not play a role in *FZD6* knockdown-induced DNA damage repair deficiency (Supplementary Fig. 2B). The RPPA assay revealed that knocking down *FZD6* downregulated WEE1 and ARID1A, both of which play a role in DNA damage repair. Besides being a critical component of the G2-M cell cycle checkpoint [30], WEE1 also maintains genomic stability by stabilizing replication forks [31] and regulate double strand DNA repair by phosphorylating and activating CDK1 [32]. The chromatin remodeler ARID1A is also a critical regulator for genome stability [33, 34]. Deficiency in ARID1A impairs the DNA damage repair pathway causing homologous recombination repair (HRR) deficiency and a BRCAness phenotype [34–37]. We evaluated whether ectopic expressions of WEE1 and ARID1A rescued the *FZD6* knockdown induced DNA damage repair deficiency. Overexpressing WEE1 to the level comparable to that in wild type DU145 cells decreased the expression of γH2AX (Fig. 4E) and the average number of γH2AX foci induced by etoposide in the DU145 cells with *FZD6* knockdown by 43.2% (Fig. 4F). Additionally, expressing WEE1 in C4-2 cells also rescued the FZD6 knockdown induced deficiency in both HR and NHEJ-mediated DNA double strand break repair as determined by the reporter assays (Fig. 4G). In contrast, although the ectopic expression of ARID1A reduced the average number of γH2AX foci by 21.8% (Supplementary Fig. 2C), it failed to rescue the DNA damage repair deficiency in the reporter assays (Supplementary Fig. 2D). These results demonstrate that WEE1, but not ARID1A, is the major DNA repair pathway component operating downstream FZD6 in regulating DNA damage repair.

WEE1 expression is tightly regulated through posttranslational modification. FZD6 knockdown did not affect the expression of *WEE1* transcript in DU145 cells (Fig. 3C). We treated the DU145 cells expressing the scrambled or *FZD6* shRNA with the protein synthesis inhibitor cycloheximide to determine WEE1 turnover. WEE1 degraded faster in the DU145 cells with *FZD6* knockdown versus the control cells (Fig. 4H). The proteosome inhibitor MG132 inhibited WEE1 turnover (Fig. 4H). These results show that FZD6 knockdown downregulates WEE1 via proteosome-mediated degradation. The M-phase kinase polo-like kinase 1 (PLK1) has been shown to phosphorylate WEE1 at S53, leading to its binding with the E3 ligase βTrCP and subsequent degradation via proteosome [38]. Knocking down *PLK1* in DU145 cells inhibited *FZD6* knockdown induced WEE1 downregulation (Fig. 4I), demonstrating that PLK1 plays a crucial role in FZD6-regulated WEE1 expression. However, neither the expression level nor cellular localization of PLK1 in DU145 cells was altered by the knockdown of *FZD6* (Supplementary Fig. 2E).

### FZD6 knockdown sensitizes prostate cancer to genotoxic stress and PKMYT1 inhibition

**Fig. 5:**
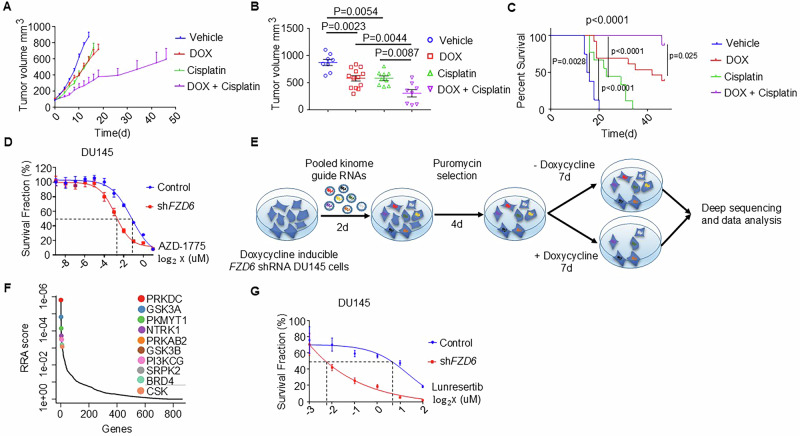
FZD6 knockdown sensitizes prostate cancers to cisplatin and lunresertib. **A** Doxycycline-induced *FZD6* knockdown in LuCaP35CR enhances therapeutic efficacy of cisplatin. Line graph shows means ± s.e.m of tumor size (*n* = 8-13) throughout the experiment. **B** Dot plot shows means ± s.e.m of tumor size in all groups when the first tumor in the control group reached 1000 mm^3^. *P* values by one-way ANOVA with Tukey’s multiple comparison test. **C** Kaplan–Meier survival analysis shows significantly better overall survival for mice in the combinatory treatment group. *p* < 0.0001, Log-rank (Mantel–Cox) test. **D** Survival curves of DU145 cells expressing scrambled and *FZD6* shRNA in presence of varying concentration of AZD-1775 by MTT assay. Survival refraction is normalized based on cells without AZD-1775 treatment. **E** Schematic illustration of experimental design for pooled kinome-wide CRISPR-Cas9 knockout screen. **F** Top genes ranked by RRA (Robust Rank Aggregation) scores using MAGeCK test. **G** Survival curves of DU145 cells expressing scrambled and *FZD6* shRNA in the presence of varying concentration of lunresertib by MTT assay. Survival fraction is normalized based on cells without lunresertib treatment.

Preclinical and clinical data have shown that WEE1 inhibition enhances the cytotoxic effect of DNA-damaging agents [39]. We showed that *FZD6* knockdown downregulated WEE1 and impaired the DNA damage repair process. Therefore, we reasoned that *FZD6* knockdown may sensitize prostate cancer cells to DNA-damaging agents. Platinum-based chemotherapy agents such as cisplatin have been used to treat advanced prostate cancers. We next evaluated whether *FZD6* knockdown enhances the therapeutic effect of cisplatin toward advanced human prostate cancers in vivo using the LuCaP35CR CRPC model that we have engineered to express the doxycycline inducible *FZD6* shRNA. By the time the volume of the tumors in the control group reached 1000 mm^3^, the average volumes of the tumors in the *FZD6* knockdown and cisplatin treatment groups were 578.3 mm^3^ and 579.9 mm^3^, respectively (Fig. 5A, B). Combinatorial treatment with *FZD6* knockdown and cisplatin further reduced the average tumor volume to 301.6 mm^3^ and significantly increased survival of the experimental mice (Fig. 5A-C). Interestingly, although *FZD6* knockdown also reduced the growth of a normal prostate cell line RWPE by 30% (Supplementary Fig. 3A), it did not sensitize RWPE to cisplatin treatment (Supplementary Fig. 3B). Collectively, these results show that targeting FZD6 enhances the therapeutic efficacy of cisplatin in castration-resistant prostate cancer.

The G1 checkpoint is dysregulated in many cancers, so inhibiting the WEE1 kinase activity is considered a promising therapeutic strategy due to its crucial role in regulating the G2-M checkpoint. As *FZD6* knockdown downregulated WEE1, we reasoned that *FZD6* knockdown may sensitize prostate cancer cells to WEE1 inhibitors. DU145 cells with *FZD6* knockdown were more susceptible to cytotoxicity induced by AZD1775, a potent inhibitor of WEE1, compared to the cells with intact FZD6 (Fig. 5D). However, the ID_50_ was enhanced by only 2-fold.

We then sought to identify other kinase targets that can cooperate with *FZD6* knockdown to inhibit prostate cancer cell growth. To this end, we performed a pooled kinome-wide CRISPR-Cas9 knockout screen in the DU145 cells expressing doxycycline-inducible *FZD6* shRNA using a lentiviral single guide RNA (sgRNA) library that contained 3052 unique sgRNAs targeting 763 human kinases (Fig. 5E). DU145 cells expressing the doxycycline-inducible *FZD6* shRNA were infected with the lentivirus expressing the kinome gRNAs at a multiplicity of infection (MOI) of 0.3. After the infected cells were selected using puromycin, they were divided into two groups and continuously cultured for 7 days in the presence and absence of doxycycline. The sgRNAs were amplified from genomic DNA and subjected to sequencing, followed by model-based analysis of genome-wide CRISPR-Cas9 knockout (MAGeCK). PKMYT1 stood up from the top identified candidates (Fig. 5F). PKMYT1 belongs to the WEE kinase family [40]. It possesses a redundant function with WEE1. PKMYT1 also phosphorylates cyclin-dependent kinase 1 (CDK1)-CyclinB1 complex to prevent cell cycle progression of cells with DNA damages. We reason that the downregulation of WEE1 by *FZD6* knockdown renders these cells more dependent on PKMYT1 for cell cycle regulation. Indeed, compared to the control cells, DU145 cells with *FZD6* knockdown display an 11-fold decrease in IC_50_ towards lunresertib, a selective inhibitor of PKMYT1 catalytic activity (Fig. 5G). This result demonstrates that lunresertib enhances the therapeutic efficacy of *FZD6*-targeted therapy.

The other two interesting candidates identified from the screen were PRKDC and PRKAB2, which encode components of the DNA-PK and AMPK complexes, respectively. However, *FZD6* knockdown did not sensitize DU145 cells to small chemical compounds interfering with the activity of these complexes (Supplementary Fig. 3C, D). This suggests that the matrix function of the kinase complexes but not their kinase activities, interact with *FZD6*-mediated signaling.

## Discussions

We show that *FZD6* is the most highly expressed and amplified frizzled receptors in both AR^+^ and AR^-^ advanced prostate cancers. We knocked down *FZD6* in multiple prostate cancer cell lines and prostate cancer patient-derived xenograft models and demonstrated that knocking down FZD6 suppresses prostate cancer growth in vitro and in vivo and attenuates the DNA damage repair process. This indicates that targeting a single FZD receptor that is highly expressed in prostate cancer cells is sufficient to achieve therapeutic efficacy, providing strong rationale for the development and application of FZD6-targeted therapies. Since *Fzd6* knockout in mice only causes a misorientation of follicle patterning [41], targeting FZD6 may avoid dose-limiting toxicity and result in a favorable therapeutic index.

The feasibility of targeting specific FZD receptors to minimize therapeutic toxicity has been demonstrated. Small-molecule inhibitors targeting FZD receptors have been reported [42], but they are only moderately specific and have never advanced to the clinical stage. Antibody-based FZD targeting is more promising. For instance, Vantictumab (OMP-18R5), a monoclonal neutralizing antibody against FZD1/2/5/7/8, has been evaluated in clinical trials for breast and pancreatic cancers [43]. OTSA101, a non-neutralizing FZD10 antibody, has been conjugated to radionuclides for the treatment of synovial sarcoma, which highly expresses FZD10 [44]. More recently, a combined computational design and experimental screening approach has been employed to generate de novo protein binders with preferential antagonism of FZD8, FZD7, and FZD4 [45]. To date, natural antibodies or protein binders that specifically antagonize FZD6 have not been reported, although artificial intelligence-assisted protein design strongly supports their feasibility. An alternative strategy for FZD6 inhibition is to conjugate siRNA targeting *FZD6* with aptamers directed against prostate cancer antigens such as PSMA (prostate-specific membrane antigen) [46] or TfR1 (Transferrin Receptor 1) [47]. Once developed, these reagents could be immediately applied in combination with lunresertib for the treatment of advanced prostate cancer based on our functional studies.

Numerous studies have shown that canonical Wnt signaling regulates DNA damage repair in different cancer models. Kaur et al. showed that using the porcupine inhibitor to block the secretion of Wnt ligands in colorectal cancer cells downregulated multiple genes in the homologous recombination and Fanconi anemia repair pathways, created a BRCAness-like state, and sensitized tumor cells to PARP inhibitors [27]. The expressions of DNA-damage repair genes were downregulated due to a blockade of the β-catenin-MYBL2 signaling axis. Similarly, depletion of FZD10 or inhibition of the Wnt/β-catenin in BRCA-mutated epithelial ovarian cancers overrode the resistance of the cells to PARP inhibitor [48]. In addition, FZD7 knockdown in ovarian cancer cells sensitized the cells to platinum-based therapy through a mechanism whereby a FZD7-β-catenin-Tp63-GPX4 pathway protected cells from chemotherapy-induced oxidative stress [49]. Finally, in triple negative breast cancer cells, FZD5 enhanced DNA damage repair by upregulating FOXM1 in a β-catenin-dependent manner [50]. Besides interacting with transcription factors such as FOXM1 and MYBL2 to regulate genes associated with DNA-damage repair and oxidative stress, β-catenin also interacts with many components of the DNA-damage repair machinery such as KU70/KU80, RAD51, and Lig4 [51, 52] or transcriptional regulators recruited by DNA damage events such as EZH2 and BRG1 [53, 54].

In contrast, how noncanonical Wnt signaling affects DNA damage repair is relatively understudied. FZD6 is unique among the frizzled receptors because it is generally considered as a noncanonical Wnt receptor, although there are also reports implying its participation in the canonical pathway. Our study using multiple prostate cancer cell lines and PDX models shows that knocking down FZD6 suppresses prostate cancer cell growth and impairs the DNA-damage repair. These results provide solid evidence that targeting noncanonical Wnt signaling can also affect the DNA damage repair process at least in the cellular contexts we investigated.

Our study reveals that knocking down FZD6 attenuates DNA-damage repair by downregulating WEE1. We showed that restoring WEE1 expression rescues the compromised DNA-damage repair resulting from FZD6 loss. We further showed that PLK1 plays an essential role in the regulation of WEE1 by FZD6. The mechanism by which FZD6 regulates PLK1 remains to be determined. The FZD6 ligand Wnt5a has been shown to promote binding of PLK1 with DVL2, thereby limiting its cytoplasmic activity [55]. Therefore, it is possible that knocking down FZD6 frees PLK1 from intracellular membrane area and enhances its interaction with and phosphorylation of WEE1. However, this hypothesis is not supported by our result showing that *FZD6* knockdown did not affect cellular localization of PLK1 (Supplementary Fig. 2F). Further study is needed to explore the underlying mechanisms. More interestingly, through the unbiased kinome gRNA screen, we revealed that downregulating FZD6 rendered the cells more sensitive to the inhibition of the other WEE kinase family member PKMYT1. The PKMYT1 inhibitor Lunresertib is already in clinical trial (NCT04855656) and shows promising therapeutic efficacy to ovarian cancer. Therefore, the therapeutic vulnerability that we uncovered may be therapeutically exploited in the clinic in the future.

Although WEE1 serves as a major downstream effector of FZD6 to regulate DNA damage repair in the cell models we evaluated, noncanonical Wnt signaling has also been shown to interact with DNA-damage repair pathways via other mechanisms. For example, another noncanonical Wnt downstream effector JNK can phosphorylate H2AX at S139 [56]. Noncanonical Wnt signaling induces epithelial-to-mesenchymal transition (EMT) and the EMT master regulator ZEB1 can promote non-homologous end joining double-strand break repair [57]. These findings and our data suggest that a more comprehensive understanding of the interaction between Wnt signaling and DNA damage repair pathway could lead to novel therapeutic strategies with better efficacy.

## Supplementary information

Supplementary Fig Legends

SF1

SF2

SF3

ST1

ST234

## Data Availability

All data generated or analyzed during this study are included in this published article and its supplementary information files.
