# Supplementary material for: Targeting FZD6 creates therapeutically actionable vulnerabilities for advanced prostate cancer
Source: Oncogene. 2025 Nov 24;44(50):4868–77. doi: 10.1038/s41388-025-03631-6 (PMC12669021; doi:10.1038/s41388-025-03631-6)
Supplement: Supplementary file 1 — Supplementary Fig Legends [file 41388_2025_3631_MOESM1_ESM.docx]

**Supplementary Fig. 1**

(A) Western blot analysis of indicated proteins in DU145 cells expressing scrambled and *FZD6* shRNAs. Three lanes for each condition represent three independent experiments. (B) Western blot analysis of indicated proteins in LuCaP176.1 cells expressing doxycycline-regulatable scrambled and *FZD6* shRNAs in the absence and presence of doxycycline (DOX).

**Supplementary Fig. 2**

(A) Western blot assay of indicated proteins in serum-starved DU145 cells expressing scrambled (shSCR) and *FZD6* shRNA (*shFZD6*). Three lanes for each condition represent three independent experiments. (B) Western blot assay of indicated proteins in DU145 cells expressing scrambled (shSCR) shRNA, *FZD6* shRNA (*shFZD6*) and *shFZD6* together with SRC or STAT3. Three lanes for each condition represent three independent experiments. (C) Immunostaining of γH2AX in DU145 cells expressing scrambled shRNA (shSCR), sh*FZD6*, and sh*FZD6* with ARID1A. Dot plot shows means ± s.d. of γ-H2AX foci per cell. N=10. P values by one-way ANOVA. (C) Dot plots show means ± s.d. of GFP^+^ C4-2 cells by flow cytometric analyses in the HR- and NHEJ- DNA double strand break repair reporter assays. Each dot shows results from one experiment. N=3. P values by two-way ANOVA with Tukey’s multiple comparison test. (D) Western blot assay of indicated proteins in DU145 cells expressing scrambled (shSCR) and *FZD6* shRNA (*shFZD6)*. T: total cell lysate, N: nuclear lysate, C: cytoplasmic lysate. Nuclear and cytoplasmic protein from equal cell numbers were loaded in each lane.

**Supplementary Fig. 3**

(A) Dot plots show means ± s.d. of survival ratio of RWPE cells expressing doxycycline-inducible scrambled or *FZD6* shRNA. Data is normalized by growth of respective control cells that were not treated with doxycycline. N=3. Student’s T-test. (B) Survival curves of RWPE cells expressing scrambled and *FZD6* shRNA in presence of varying concentration of Cisplatin by MTT assay. Survival fraction is calculated by normalization with cells without Cisplatin treatment. (C-D) Survival curves of DU145 cells expressing scrambled or *FZD6* shRNA in presence of varying concentration of the DNK-PK inhibitor (AZD-7648) and mTOR inhibitor (AZD-8055) by MTT assay. Survival fraction is calculated based on cells without drug treatment.
