# Supplementary figures and images for: Targeting FZD6 creates therapeutically actionable vulnerabilities for advanced prostate cancer
Source: Oncogene. 2025 Nov 24;44(50):4868–77. doi: 10.1038/s41388-025-03631-6 (PMC12669021; doi:10.1038/s41388-025-03631-6)

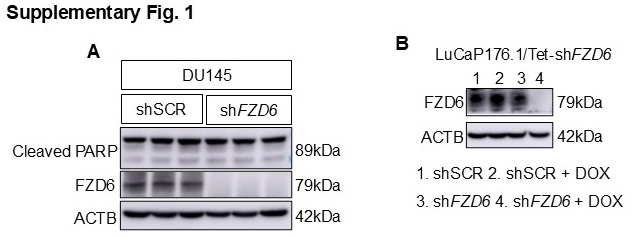

Supplement: Supplementary file 2 — SF1 [file 41388_2025_3631_MOESM2_ESM.jpg]

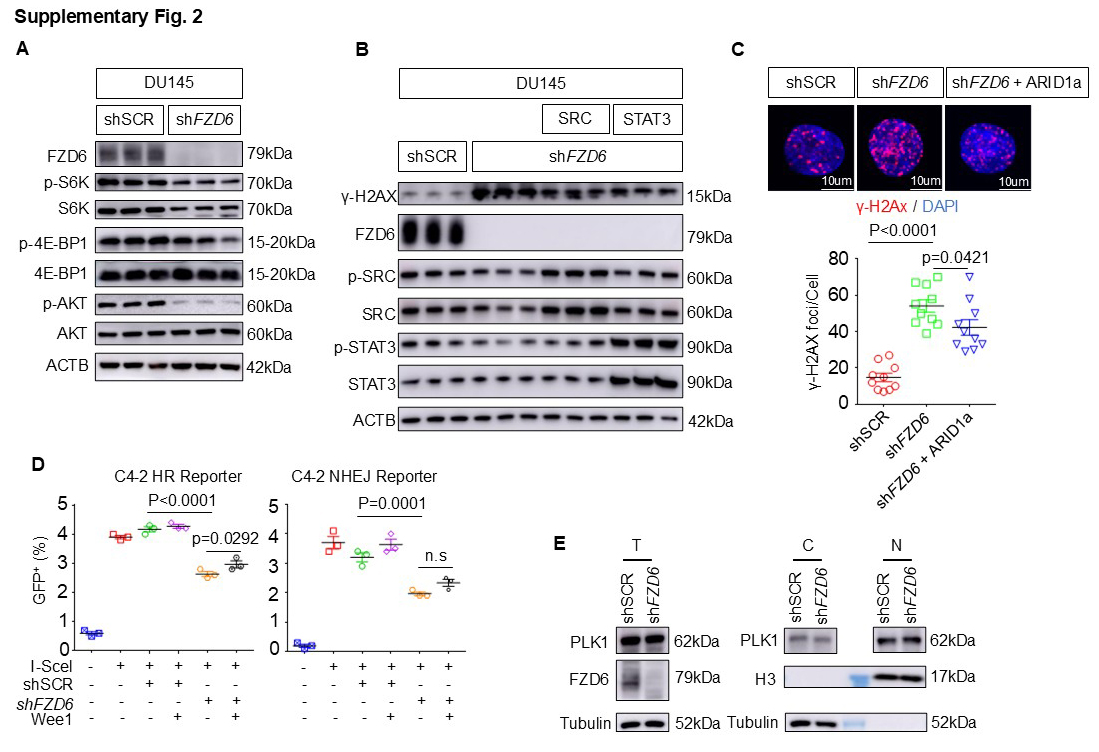

Supplement: Supplementary file 3 — SF2 [file 41388_2025_3631_MOESM3_ESM.jpg]

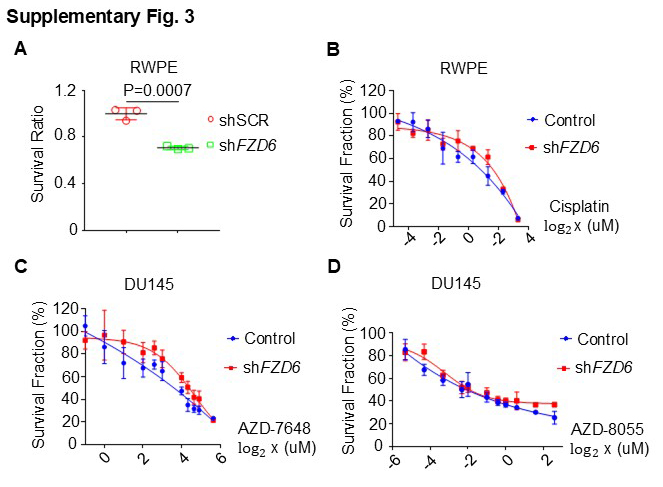

Supplement: Supplementary file 4 — SF3 [file 41388_2025_3631_MOESM4_ESM.jpg]
