# Supplementary material for: Targeting FZD6 creates therapeutically actionable vulnerabilities for advanced prostate cancer
Source: Oncogene. 2025 Nov 24;44(50):4868–77. doi: 10.1038/s41388-025-03631-6 (PMC12669021; doi:10.1038/s41388-025-03631-6)
Supplement: Supplementary file 6 — ST234 [file 41388_2025_3631_MOESM6_ESM.docx]

**Supplementary Table 2: qRT-PCR primers**

| Primers | Sequences (5’ to 3’) |
| --- | --- |
| *FZD1* forward | ATCTTCTTGTCCGGCTGTTACA |
| *FZD1* reverse | GTCCTCGGCGAACTTGTCATT |
| *FZD2* forward | GTGCCATCCTATCTCAGCTACA |
| *FZD2* reverse | CTGCATGTCTACCAAGTACGTG |
| *FZD3* forward | GTTCATGGGGCATATAGGTGG |
| *FZD3* reverse | GCTGCTGTCTGTTGGTCATAA |
| *FZD4* forward | CCTCGGCTACAACGTGACC |
| *FZD4* reverse | TGCACATTGGCACATAAACAGA |
| *FZD5* forward | CATGCCCAACCAGTTCAACC |
| *FZD5* reverse | CGGCGAGCATTGGATCTCC |
| *FZD6* forward | ATGGCCTACAACATGACGTTT |
| *FZD6* reverse | GTTTACGACAAGGTGGAACCA |
| *FZD7* forward | GTGCCAACGGCCTGATGTA |
| *FZD7* reverse | AGGTGAGAACGGTAAAGAGCG |
| *FZD8* forward | ATCGGCTACAACTACACCTACA |
| *FZD8* reverse | GTACATGCTGCACAGGAAGAA |
| *FZD9* forward | TGCGAGAACCCCGAGAAGT |
| *FZD9* reverse | GGGACCAGAACACCTCGAC |
| *FZD10* forward | AGCCATCCAGTTGCACGAG |
| *FZD10* reverse | GAGTCGGGCCACTTGAAGTT |
| *ARID1a* forward | CAGTACCTGCCTCGCACATA |
| *ARID1a* reverse | GCCAGGAGACCAGACTTGAG |
| *KMD5B* forward | CCATAGCCGAGCAGACTGG |
| *KMD5B* reverse | GGATACGTGGCGTAAAATGAAGT |
| *KMT2A* forward | AAGAGCAGGTAAACTCTCTCCTC |
| *KMT2A* reverse | TTCCTCTCCGTCGTACAATTTG |
| *KMT2D* forward | GAGCTACGGCGCTTTGAGTT |
| *KMT2D* reverse | AGGGAAACCAATCTGTGATAGGT |
| *POLE* forward | TTCCTCAGTTTCGGCACTCAA |
| *POLE* reverse | CTCAAAACCAAACCGCAAATCC |
| *ENDOV* forward | CACTGTGGAAACGGGAGCAA |
| *ENDOV* reverse | TTCACGAAGGACACGTCAACG |
| *XPC* forward | CTTCGGAGGGCGATGAAAC |
| *XPC* reverse | TTGAGAGGTAGTAGGTGTCCAC |
| *WEE1* forward | AGGGAATTTGATGTGCGACAG |
| *WEE1* reverse | CTTCAAGCTCATAATCACTGGCT |
| *GAPDH* forward | ATTGCCCTCAACGACCACT |
| *GAPDH* reverse | ATGAGGTCCACCACCCTGT |

**Supplementary Table 3: Primers for cloning**

| Primer | Forward | Reverse |
| --- | --- | --- |
| FZD6 ORF-XbaI | AAATCTAGAGCCACCATGGAAATGTTTAC | TTTTCTAGAAATCAAGTATCTGAATGACAACCAC |
| shRNA-FZD6 #1 | CCGGCCACCCATTGATTGTATTATACTCGAGTATAATACAATCAATGGGTGGTTTTTG | AATTCAAAAACCACCCATTGATTGTATTATACTCGAGTATAATACAATCAATGGGTGG |
| shRNA-FZD6 #2  (Mostly commonly used shRNA) | CCGGACCCAGAGAGACCAATTATATCTCGAGATATAATTGGTCTCTCTGGGTTTTTTG | AATTCAAAAAACCCAGAGAGACCAATTATATCTCGAGATATAATTGGTCTCTCTGGGT |
| Tet on inducible shRNA sequence | CCGGACCCAGAGAGACCAATTATATCTCGAGATATAATTGGTCTCTCTGGGTTTTTTG | AATTCAAAAAACCCAGAGAGACCAATTATATCTCGAGATATAATTGGTCTCTCTGGGT |
| PLKO.1 shRNA scramble sequence & tet on inducible scramble sequence | CCGGCCTAAGGTTAAGTCGCCCTCGCTCGAGCGAGGGCGACTTAACCTTAGGTTTTTG | AATTCAAAAACCTAAGGTTAAGTCGCCCTCGCTCGAGCGAGGGCGACTTAACCTTAGG |
| Inducible Wee1 ORF (ORF was cloned by NheI and SalI into PCW57.1 Vector) | GCTAGCATGGATACAGAAAAATCAGGAAAAAG | GTCGACTCACTTGTCATCGTCATCCTTGTAGTCGATGTCATGATCTTTATAATCACCGTCATGGTCTTTGTAGTCGTATATAGTAAGGCTGACAGAG |
| shRNA-PLK1 | CCGGCGATACTACCTACGGCAAATTCTCGAGAATTTGCCGTAGGTAGTATCGTTTTTG | AATTCAAAAACGATACTACCTACGGCAAATTCTCGAGAATTTGCCGTAGGTAGTATCG |

**Supplementary Table 4: Antibodies for Western blot and immunostaining**

| Antigen | Supplier | Cat. Number/Clone Number | Species | Dilution |  |
| --- | --- | --- | --- | --- | --- |
| FZD6 | Cell Signaling | 5158S | Rabbit | 1:1000 |  |
| γ-H2AX | Cell Signaling | 9718S | Rabbit | 1:1000 |  |
| WEE1 | Cell Signaling | 4936S | Rabbit | 1:1000 |  |
| SRC | Cell Signaling | 2108S | Rabbit | 1:1000 |  |
| p-SRC | Cell Signaling | 6943S | Rabbit | 1:1000 |  |
| AKT | Cell Signaling | 2938P | Rabbit | 1:1000 |  |
| p-AKT | Cell Signaling | 4060S | Rabbit | 1:1000 |  |
| STAT3 | Cell Signaling | 8768S | Rabbit | 1:1000 |  |
| p-STAT3 | Cell Signaling | 4113S | Rabbit | 1:1000 |  |
| PLK1 | Cell Signaling | 4535S | Rabbit | 1:1000 |  |
| Caspase 3 | Cell Signaling | 9661S | Rabbit | 1:1000 |  |
| Cleaved PARP | Cell Signaling | 5625T | Rabbit | 1:1000 |  |
| BrdU | Abcam | ab6326 | Rat | 1:500 |  |
| β-actin | Sigma | A2228 | Mouse | 1:1000 |  |
| Tubulin | | Cell Signaling | 2144S | Rabbit | 1:1000 |
| H3 | | Cell Signaling | 9715S | Rabbit | 1:1000 |
| S6K | | Cell Signaling | 9202S | Rabbit | 1:1000 |
| p-S6K | | Cell Signaling | 9234S | Rabbit | 1:1000 |
| 4E-BP1 | | Cell Signaling | 9644S | Rabbit | 1:1000 |
| p-4E-BP1 | | Cell Signaling | 9451S | Rabbit | 1:1000 |
